# Activators and Effectors of the Small G Protein Arf1 in Regulation of Golgi Dynamics During the Cell Division Cycle

**DOI:** 10.3389/fcell.2018.00029

**Published:** 2018-03-26

**Authors:** Catherine L. Jackson

**Affiliations:** Institut Jacques Monod, Centre Nationnal de la Recherche Scientifique, UMR 7592, Université Paris Diderot, Sorbonne Paris Cité, Paris, France

**Keywords:** ADP-ribosylation factor (Arf), guanine nucleotide exchange factor, Golgi brefeldin A resistance factor 1 (GBF1), cell division cycle, mitosis, Golgi, endoplasmic reticulum, self organization

## Abstract

When eukaryotic cells divide, they must faithfully segregate not only the genetic material but also their membrane-bound organelles into each daughter cell. To assure correct partitioning of cellular contents, cells use regulatory mechanisms to verify that each stage of cell division has been correctly accomplished before proceeding to the next step. A great deal is known about mechanisms that regulate chromosome segregation during cell division, but we know much less about the mechanisms by which cellular organelles are partitioned, and how these processes are coordinated. The Golgi apparatus, the central sorting and modification station of the secretory pathway, disassembles during mitosis, a process that depends on Arf1 and its regulators and effectors. Prior to total disassembly, the Golgi ribbon in mammalian cells, composed of alternating cisternal stacks and tubular networks, undergoes fission of the tubular networks to produce individual stacks. Failure to carry out this unlinking leads to cell division arrest at late G2 prior to entering mitosis, an arrest that can be relieved by inhibition of Arf1 activation. The level of active Arf1-GTP drops during mitosis, due to inactivation of the major Arf1 guanine nucleotide exchange factor at the Golgi, GBF1. Expression of constitutively active Arf1 prevents Golgi disassembly, and leads to defects in chromosome segregation and cytokinesis. In this review, we describe recent advances in understanding the functions of Arf1 regulators and effectors in the crosstalk between Golgi structure and cell cycle regulation.

## Introduction

Arf proteins are small GTP-binding (G) proteins that are regulated through a cycle of GTP binding and hydrolysis (Gillingham and Munro, 2007; Donaldson and Jackson, 2011). In their active GTP-bound form, Arf proteins are tightly associated with the membrane bilayer. Hence they bring their effectors, proteins that bind specifically to the GTP-bound form, into close contact with the lipid bilayer. The Arf proteins are part of a larger family that also includes the Arf-like (Arl) proteins, whose diverse functions include membrane trafficking, targeting of proteins to cilia, microtubule regulation, and lysosome function (Gillingham and Munro, 2007; Donaldson and Jackson, 2011). Arf and many Arl proteins are modified by addition of a hydrophobic myristoyl group to the amino-terminal amphipathic helix. When GTP binds an Arf protein, this myristoylated helix inserts into the membrane, mediating membrane association (Antonny et al., 1997; Pasqualato et al., 2002).

There are three classes of mammalian Arf proteins, Class I (Arfs1-3), Class II (Arfs 4-5), and Class III (Arf6), the division being largely based on sequence homology. Class I Arfs are highly conserved in evolution, are present in all eukaryotes, and play an essential role at the Golgi in the secretory pathway (Manolea et al., 2010; Donaldson and Jackson, 2011). Class III Arfs are also widespread among eukaryotes, but are less conserved than Class I Arfs, and function at the cell periphery (Donaldson and Jackson, 2011). The Class II Arfs are found exclusively in the animal lineage, arising relatively late in evolution. Although absent from fungi, Class II Arfs are present in the free-living unicellular flagellates, the choanoflagellates (Manolea et al., 2010; Schlacht et al., 2013).

**Figure 1:**
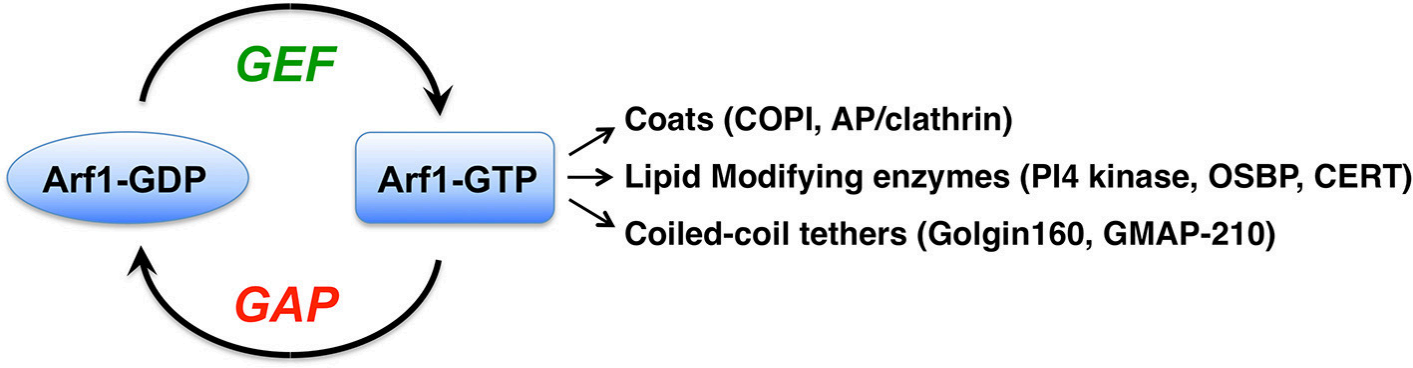
The Arf1 G protein is activated by guanine nucleotide exchange factors (GEFs) through their catalysis of GDP nucleotide release from Arf. Binding of the more abundant GTP in cells leads to conformational changes in the small G protein, allowing it to interact with numerous proteins called effectors. The major classes of Arf1 effectors are listed, and a few examples of each are indicated in parentheses. COPI, coat protein complex I; AP, adaptor protein; PI4 kinase, phosphatidylinositol-4-kinae; OSBP, oxysterol binding protein; CERT, ceramide transfer protein.

The spatio-temporal control of Arf protein function is mediated by regulators of Arf-GTP binding and GTP hydrolysis. The Arf guanine nucleotide exchange factors (GEFs) catalyse GDP release from their substrate Arf, which results in GTP binding due to the large excess of GTP over GDP in cells (Figure 1). This nucleotide exchange activity is carried out by the Sec7 domain, an evolutionarily conserved sequence first identified as a homology domain in the yeast Sec7p protein (Achstetter et al., 1988). There are seven subfamilies of Arf GEFs in eukaryotic cells (Cox et al., 2004). Members of the GBF/Gea and BIG/Sec7 subfamilies of Sec7 domain GEFs use Class I Arf proteins as substrates, and GBF1 proteins may also use Class II Arfs as substrates (Donaldson and Jackson, 2011). These two subfamilies have a similar domain structure, likely due to a common ancestor, but have different steady-state localizations and functions (Casanova, 2007; Bui et al., 2009; Donaldson and Jackson, 2011). Among all of the Sec7 domain GEFs, members of only the GBF/Gea and BIG/Sec7 subfamilies of Arf GEFs are sensitive to the fungal toxin brefeldin A (BFA), which acts as an uncompetitive inhibitor that stabilizes an inactive GEF-Arf1-GDP complex, thus blocking Arf1 activation (Peyroche et al., 1999; Robineau et al., 2000; Mossessova et al., 2003). The function of the Sec7 domain was first identified in yeast Gea1p (Peyroche et al., 1996) and mammalian Arf nucleotide-binding site opener (ARNO) (Chardin et al., 1996). The human orthologue of yeast Gea1p, Golgi brefeldin A resistance factor 1 (GBF1), was identified by Paul Melançon and colleagues as a Golgi-localized protein whose overexpression conferred resistance to BFA (Mansour et al., 1998; Claude et al., 1999).

## Arf1 effectors: downstream consequences of Arf1 activation

Arf1 plays a major role in maintaining the structure and function of the Golgi apparatus. Arf1 localizes throughout the Golgi, as well as to the ER-Golgi intermediate compartment (ERGIC) and to recycling endosomes (Donaldson and Jackson, 2011; Bottanelli et al., 2017). Active GTP-bound Arf1 recruits numerous coats to different membrane sites (Donaldson and Jackson, 2011; Bonifacino, 2014) (Figure 1). The COPI coat is recruited by Arf1 to ERGIC and Golgi membranes, where it mediates retrograde trafficking back to the ER or to an earlier Golgi compartment (Donaldson and Jackson, 2011; Jackson, 2014). Arf1 and Arf3 at the *trans*-Golgi and *trans*-Golgi network (TGN) recruit several coat complexes, including GGA1-3/clathrin, AP-1/clathrin, AP-3/clathrin, and AP-4 coats (Bonifacino and Glick, 2004; Bonifacino, 2014). Arf1 activation also leads to changes in the membrane lipids themselves through Arf1-GTP recruitment of lipid modifying enzymes (Donaldson and Jackson, 2011).

An important class of Arf1 effectors comprises the long coiled-coil Golgi tethering proteins, including golgin160 and GMAP-210 (Gillingham et al., 2004; Ríos et al., 2004; Drin et al., 2008; Yadav et al., 2012). GMAP-210 is a tether that links small ER-Golgi vesicles (both COPI and COPII) to flat Golgi cisternae (Drin et al., 2008; Wong and Munro, 2014), and also mediates Golgi-centrosome association (Ríos et al., 2004). Cells depleted of GMAP-210 undergo Golgi fragmentation (Ríos et al., 2004). Golgin160 serves as a membrane receptor for cytoplasmic dynein-1 (hereafter referred to as dynein) at the Golgi, through a direct interaction with the dynein intermediate chain (Yadav et al., 2012). Dynein is the major minus-end directed microtubule motor in eukaryotic cells, using the energy from ATP hydrolysis to drive movement of cargo along microtubule tracks (Vale, 2003; Carter, 2013). Composed of a homodimer of a motor domain-containing heavy chain and several additional subunits, dynein serves multiple functions in cells (Vallee et al., 2012; Cianfrocco et al., 2015; Bhabha et al., 2016). GBF1 activation of Arf1 is required for golgin160 binding to *cis*-Golgi membranes, as golgin160 is no longer present on Golgi membranes in cells treated with GBF1 siRNA or treated with the GBF/BIG-specific inhibitor BFA (Yadav et al., 2012). In this manner, GBF1 and Arf1 regulate movement of Golgi elements on microtubules, which is required to maintain the positioning of the Golgi ribbon close to the centrosome at the cell center, and is a prerequisite for linking of the Golgi ribbon into one continuous structure.

## Changes in golgi morphology during the cell cycle

Dividing cells must exactly duplicate their chromosomal DNA, and segregate each copy into the two daughter cells (Hartwell and Weinert, 1989). The demarcation of the cell division cycle into four phases is based on the major steps in transmission of the genetic material during cell division. The first phase, G1, represents the initial gap phase after cells are born and before they begin DNA replication. Synthesis of DNA during the DNA replication phase (S) is followed by a second gap phase (G2). After G2, cells enter the mitotic phase (M), where the duplicated chromosomes are separated and which culminates in cytokinesis. In addition to duplication and segregation of chromosomes, many other cellular components must also be precisely divided into the two daughter cells, including the centrosome, mitochondria, the ER, Golgi, endosomes, and lysosomes (Warren and Wickner, 1996).

**Figure 2:**
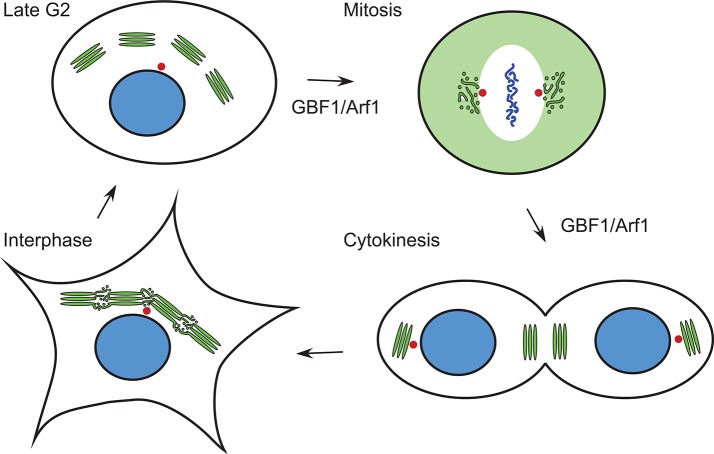
Changes in Golgi morphology during the cell cycle. The Golgi ribbon is composed of saccular regions interconnected by tubulo-vesicular zones in interphase. In late G2, the saccular Golgi regions are separated at the level of the tubular-vesiculare regions. In metaphase and anaphase of mitosis, Golgi proteins are found in either a diffuse pattern, sometimes corresponding to ER localization (e.g., ERGIC53, see Figure 3), or in tubular-vesicular clusters associated with the centrosome. During cytokinesis, two Golgi elements reform in each daughter cell, one near the midbody, the other near the centrosome. Green, Golgi proteins; blue, nucleus/chromosomes; red, centrosomes. Stages of the cell cycle at which GBF1 and Arf1 are known to function are indicated.

The Golgi apparatus forms a continuous ribbon that is centrally located near the centrosome in mammalian cells (Rambourg and Clermont, 1990; Gosavi and Gleeson, 2017). During mitosis, the Golgi undergoes a dramatic disassembly, which occurs in at least two steps (Altan-Bonnet et al., 2004; Sengupta et al., 2015; Valente and Colanzi, 2015; Wei and Seemann, 2017; Figure 2). In G2, the interlinking of stacked Golgi cisternae within the ribbon is broken, leading to their separation into individual units (Tang and Wang, 2013; Valente and Colanzi, 2015; Rabouille and Linstedt, 2016). By metaphase, the Golgi undergoes cisternal unstacking and vesiculation. Golgi enzymes and other ERGIC and Golgi proteins show a homogeneous dispersed pattern by light microscopy (Zaal et al., 1999; Altan-Bonnet et al., 2006; Marie et al., 2012). Other Golgi proteins such as the golgin GM130 show a pattern of small, dispersed puncta, whose abundance depends on the activity of the mitotic kinase Plk1 (Preisinger et al., 2005). The Rab1 small G protein associates with a tubule-vesicular compartment that is associated with the centrosome (Marie et al., 2012). In late mitosis, the Golgi reforms. Initially two Golgi elements appear, one near the cytokinesis ring, the other near the newly formed centrosome (Altan-Bonnet et al., 2004; Valente and Colanzi, 2015). In late anaphase, the smaller Golgi element moves in a MT-dependent manner toward the larger, centrosome-proximal Golgi element, and the two merge (Valente and Colanzi, 2015; Figure 2).

## Functions of Arf1 during mitosis

Arf1 levels fluctuate during the cell cycle, with important functional consequences. When cells enter prophase, the amount of membrane-bound active Arf1-GTP decreases and the cytosolic GDP-bound pool increases (Altan-Bonnet et al., 2003; Morohashi et al., 2010; Mao et al., 2013). The lowest level of Arf1-GTP is reached in metaphase, then levels increase again as cells progress through telophase (Altan-Bonnet et al., 2003; Morohashi et al., 2010). The decreased Arf1-GTP levels are due to decreased activity of the major Golgi Arf1 exchange factor, GBF1, which is phosphorylated in mitosis by AMP-activated protein kinase (AMPK) and cyclin-dependent kinase 1 (CDK1) (Morohashi et al., 2010; Mao et al., 2013). This mitotic phosphorylation of GBF1 leads to its release from membranes and a decrease in its capacity to activate Arf1. In support of the idea that there is a functional importance to the drop in Arf1-GTP during mitosis, expression of constitutively active Arf1-Q71L prevents normal mitotic Golgi disassembly, and leads to defects in chromosome segregation and cytokinesis furrow ingression (Altan-Bonnet et al., 2003). Inactivation of Arf1 contributes to Golgi disassembly through inhibition of Arf1 effector recruitment to membranes. However, Arf1 activation by GBF1 is not completely blocked during mitosis (Morohashi et al., 2010). It is not clear whether there are spatially or temporally restricted regions of complete inhibition of Arf1 activation, or whether there is only a partial inhibition throughout the entire cell during all stages of mitosis. This is an important open question to address in future studies.

**Figure 3:**
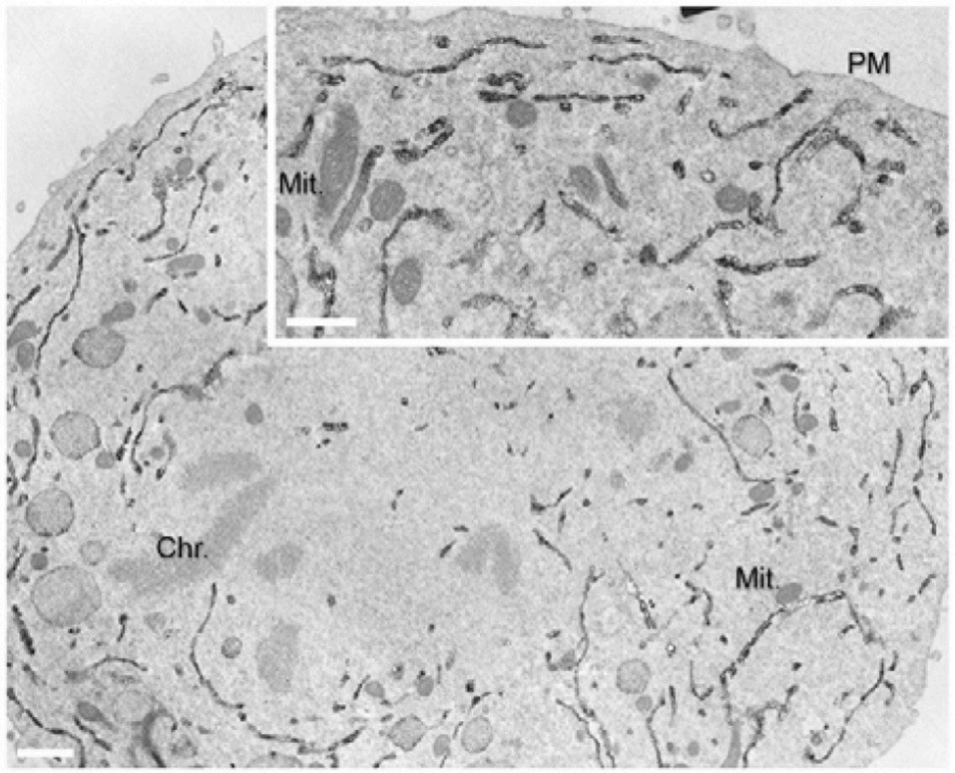
Localization of ERGIC53/p58 to the ER during anaphase. Immunoperoxidase EM localisation of p58 was performed on normal rat kidney cells. Reproduced with permission from Marie et al. (2012).

There are several effectors of GBF1 and Arf1 whose role in mitotic Golgi disassembly has been demonstrated. *In vitro* reconstitutions have shown that COPI vesicles can continue to bud in mitotic extracts, but fusion is blocked (Misteli and Warren, 1994; Sonnichsen et al., 1996; Shorter and Warren, 2002). COPI has been shown to function during mitosis in the redistribution of ERGIC53 and mannosidase II from the ERGIC and Golgi to their dispersed metaphase pattern (Marie et al., 2012). In the case of ERGIC53, this dispersed pattern is a result of relocation of the protein to the ER (Marie et al., 2012; Figure 3). These results are based on treatment of cells with the GBF1 inhibitor BFA, which completely blocks COPI recruitment to membranes (Orci et al., 1991; Klausner et al., 1992). Hence retrograde transport mediated by COPI persists during mitosis, at least to some extent.

Golgin160, an effector of Arf1 that recruits the dynein motor to early Golgi membranes, is an effector of Arf1 which function in mitotic Golgi disassembly. Golgin160 is released from Golgi membranes in mitosis as a direct consequence of Arf1 inactivation, leading to loss of dynein from these membranes, which correlates temporally with the Golgi apparatus becoming fragmented (Yadav et al., 2012). The role of the Arf1 effector and membrane tether GMAP-210, has not been explored in mitosis. However, as GMAP-210 has an N-terminal motif that binds to COPI and COPII vesicles, and is involved in Golgi positioning near the centrosome, studying it's role during mitosis may shed new light on mechanisms of Golgi disassembly and reassembly during the cell cycle.

The final class of Arf1 effectors, lipid modifying enzymes, includes FAPP2, CERT, and OSBP. These lipid transfer proteins function at ER-Golgi membrane contact sites, where they transfer glucosylceramide, ceramide and sterol, respectively. Each of these proteins have a PH domain that requires both Arf1 and PI4P in order to bind to *trans*-Golgi membranes (De Matteis and Godi, 2004). The fate of ER-Golgi membrane contact sites during the cell cycle has not been explored, and their role in maintaining the lipid composition of the ER-Golgi system during the cell cycle is another important unanswered question.

## Coordination of golgi, centrosome, and microtubule dynamics during mitosis

The results described in the previous section indicate that activation of Arf1 by GBF1 plays a key role in regulation of Golgi structure and partitioning during mitosis. Significant recent progress has been made in unveiling the signaling pathways connecting Golgi structure to cell cycle progression, and intriguingly, the data point to a role for Arf1 activity in these pathways. **M**APK/**E**xtracellular signal regulated kinase (ERK) **K**inase MEK1 is the second of three kinases in the mitogen-activated protein kinase (MAPK) cascade, which functions in cell proliferation, survival and differentiation (Roberts and Der, 2007). MEK1 inhibition leads to a delay at the G2/M transition in the cell cycle (Feinstein and Linstedt, 2007). Inactivation of Arf1 using BFA, or deletion of GRASP65, can bypass this G2/M delay (Feinstein and Linstedt, 2007). Inhibition of Src kinases also leads to a potent G2 arrest (Roche et al., 1995). Antonino Colanzi and colleagues have shown that activation of a pool of Src kinase at the Golgi is responsible for phosphorylation of Aurora A and its recruitment to centrosomes, where it mediates centrosome maturation prior to entry into mitosis (Valente and Colanzi, 2015). BFA treatment for only 5 min, which acutely decreases the level of Arf1-GTP, blocks the activation of Src and Aurora A, thus preventing pre-mitotic centrosome maturation (Valente and Colanzi, 2015). These results support the conclusion that perturbations of Golgi structure, through different mechanisms including Arf1 inactivation, are transmitted to kinases that regulate cell cycle progression. The nature of the signaling pathways upstream of MEK1 and Src kinases at the Golgi, and how these pathways are integrated with Arf1 and downstream effectors, are important open questions.

The fact that the Src-Aurora A signaling pathway makes centrosome maturation dependent on Golgi disassembly provides an example of how the inheritance of two organelles in the cell is coordinated. Further insight into this Golgi-centrosome interrelationship has been uncovered in a recent study (Guizzunti and Seemann, 2016). When cells expressing the medial Golgi enzyme sialyltransferase fused to horseradish peroxidase (HRP) were treated with 3,3′-diaminobenzidine (DAB), the action of luminal HRP produced large DAB polymers within the Golgi that prevented normal Golgi dynamics, including its disassembly during mitosis. However, unlike the situation for other perturbations that block Golgi fragmentation, the DAB polymer-containing cells were not arrested in G2, but were blocked in mitosis with the spindle assembly checkpoint (SAC) active (Guizzunti and Seemann, 2016). The SAC is a surveillance system that monitors spindle formation and kinetochore attachment to spindle microtubules (Musacchio, 2015). In these cells, the centrosomes fail to undergo separation, thus preventing spindle formation and triggering the SAC (Guizzunti and Seemann, 2016). Remarkably, treatment with centrinone, which depletes cells of centrosomes (Wong et al., 2015), bypassed the mitotic block and restored cell growth by allowing spindle formation through an alternative microtubule nucleating mechanism (Guizzunti and Seemann, 2016). Although the mechanism has not been defined, these results show that normal Golgi dynamics and structure are important for spindle formation.

Src kinases also regulate the cycling of Golgi glycosylation enzymes between the Golgi and the ER in an Arf1-dependent manner. Activation of Src promotes the redistribution of the N-acetylgalactosaminyl transferases (GalNac-Ts) from the Golgi to the ER through upregulation of their transport into COPI vesicles (Gill et al., 2010). GalNac-Ts initiate O-glycosylation of proteins, and their presence in the ER leads to abnormal glycosylation, which is a hallmark of a number of cancers, notably breast cancer (Cazet et al., 2010). Breast cancer cells frequently have an increase in short *O*-glycans on proteins such as mucins due to the presence of GalNac-Ts in the ER, which alter their cell adhesion and migration properties and are associated with a poor prognosis (Brockhausen, 2006; Cazet et al., 2010). Frederic Bard and colleagues found that all GalNac-Ts tested were redistributed from the Golgi to the ER upon activation of Src, whereas other Golgi glycosylation enzymes, including mannosidase II, β4-galatosyltransferase and glucosaminyl*N*-acetyl transferase 1 core 2, were not affected by Src and maintained their normal Golgi localization (Gill et al., 2010). Interestingly, the glycosylation enzymes not affected by Src activation are redistributed to the ER upon BFA treatment, which inhibits COPI vesicle formation. A recent paper has challenged the redistribution of GalNac-Ts from the Golgi to the ER presented in Gill et al. 2010 (Herbomel et al., 2017), but caution in interpreting results due to various factors such as the difficulty in detecting proteins when they are diluted throughout the ER, is warranted (Bard and Chia, 2017). Given the frequent presence of hyperactive Src in cancer cell lines, understanding how Src regulates glycosylation enzyme cycling between the Golgi and ER, and how this regulation is coordinated with the Src-Aurora A Golgi-centrosome maturation pathway during the cell cycle, are important areas to explore in the future.

## Golgi structure and cell cycle regulatory pathways

Actin and the Golgi tethering proteins GRASP65 and GRASP55 are involved in linking individual Golgi stacks in the tubular network regions that connect the compact saccular zones of the Golgi (Puthenveedu et al., 2006; Feinstein and Linstedt, 2008; Rabouille and Linstedt, 2016). Both GRASP65 and GRASP55 are phosphorylated by cell cycle-regulated kinases, including Polo kinase (PLK1), CDK1 and ERK, and failure to do so results in cell cycle arrest in late G2 phase (Sutterlin et al., 2002; Rabouille and Kondylis, 2007; Rabouille and Linstedt, 2016). Aspects of this cell cycle regulation are conserved in evolution. In most Drosophila cells, the Golgi does not form one continuous ribbon, but rather pairs of Golgi stacks are linked together by F-actin and associated binding proteins (Kondylis et al., 2007). This actin-mediated linking of Golgi stacks is disrupted in late G2, and failure to separate the paired Golgi stacks leads to a cell cycle arrest prior to entry into mitosis (Kondylis et al., 2007). In yeast, the Golgi has a different structure, composed not of stacks but of multiple, scattered tubular network structures, likely corresponding to the tubular networks linking the Golgi stacks in mammalian cells (Jackson, 2009). Despite this different morphology, yeast have a GRASP orthologue, Grh1 (Behnia et al., 2007). Remarkably, when Grh1 is deleted, cells fail to undergo SAC-mediated mitotic arrest in the presence of the MT-depolymerizing drug benomyl, a function elucidated through identification of a peptide inhibitor of the kinase Mps1 (Norman et al., 1999). Mps1 overexpression leads to phosphorylation of Mad1, which triggers the SAC, even in the absence of spindle or kinetochore attachment defects (Hardwick et al., 1996). Grh1 was identified as the major target of this Mps1 inhibitor (Norman et al., 1999). Interestingly, Mps1 also plays a key role in centrosome duplication and maturation (Liu and Winey, 2012), and hence these results in yeast point to another level of co-regulation of Golgi and centrosome inheritance in eukaryotic cells.

Much effort has been devoted to understanding the functions of the mammalian GRASP55 and 65 proteins in Golgi unlinking during mitosis, but the studies in yeast described above suggest that these proteins may have functions in mitosis other than simple separation of Golgi stacks. The fact that the Drosophila orthologue of GRASPs has functions other than linking of Golgi stacks (Kondylis et al., 2007) supports this conclusion. On the other hand, the roles during mitosis of other proteins involved in maintaining a linked Golgi ribbon have not been well explored. As described above, the microtubule minus-end directed motor dynein is required to maintain the continuous centrosome-proximal Golgi ribbon structure through GBF1 and Arf1 regulation of golgin160 (Yadav et al., 2012). Hence the maintenance of a continuous Golgi ribbon is a dynamic process, involving not only Golgi tethers, but also the actin cytoskeleton and movement of Golgi elements along microtubules. Dynamic cycling of Golgi glycosylation enzymes may be an additional pathway tied into these processes of Golgi ribbon maintenance, in light of recent results on GOLPH3, an actin cytoskeleton regulator. GOLPH3 (yeast Vps74) regulates the cycling of glycosylation enzymes between the ER and Golgi in both yeast and mammalian cells (Schmitz et al., 2008; Buschman et al., 2015). Recently, GOLPH3 has been reported to function in the unlinking of Golgi stacks in mammalian cells in response to activation of the DNA damage checkpoint (Farber-Katz et al., 2014). This checkpoint normally blocks the cell cycle in G2, but cells can adapt and resume growth upon DNA damage (Ciccia and Elledge, 2010). GOLPH3 is phosphorylated directly by the DNA damage kinase DNA-PK, which leads to Golgi fragmentation and promotes survival and proliferation of cells after they have undergone DNA damage (Farber-Katz et al., 2014). It is currently unknown whether this regulation has any overlap with the pathway that promotes phosphorylation of GRASP55 and GRASP65, Golgi unlinking and relief of a G2 cell cycle arrest. However, an interesting hypothesis is that DNA-PK phosphorylation of GOLPH3 lies upstream of GRASP-mediated unlinking of the Golgi ribbon.

## Conclusions

Organelles are not static structures, but are components of dynamic membrane systems. The structures within cells that we see, at a given place and a given time, are the result of underlying dynamic processes. Evidence presented in this review suggests that the Golgi is a self-organizing structure that arises from the ER and is maintained by a constant flux of proteins and lipids through it. During mitosis, regulation of the equilibrium between anterograde and retrograde pathways tips the balance to cause redistribution of Golgi components back to the ER. The Arf1 small G protein and its activator GBF1 regulate Golgi-ER retrograde trafficking during mitosis and in cancer cells, at least in part through phosphorylation of GBF1 by mitotic kinases. Recent results connecting cell cycle regulation to Golgi morphology, a process that depends on Arf1-mediated dynein recruitment to Golgi membranes, poses new questions and opens up new perspectives for understanding the integration of organelle and chromosome segregation pathways during mitosis. Src kinase activation and the DNA damage checkpoint both impact Golgi linking and G2 arrest. Golgi fragmentation is also co-regulated with centrosome maturation and duplication, but the signals at the level of the Golgi involved in this regulation have not been determined.

The mechanisms by which organelles are partitioned during mitosis, and how these pathways are coordinated with chromosome segregation, are important questions. Cancer cells must succeed in overriding all checkpoints and blocks to cell division, and recent results have provided new information on the relevance of organelle segregation for cancer cells (Asare et al., 2017). Moreover, it is already known that an inhibitor of a key cell cycle kinase, Mps1, targets a *cis*-Golgi protein in yeast (Norman et al., 1999), but little is know about the mechanisms involved. Hence understanding how Golgi membranes are reorganized during mitosis, and the functions of the key *cis*-Golgi regulators GBF1 and Arf1 in this process, are important not only for understanding the fundamental principals of cellular organization but also in developing therapeutic approaches for the treatment of diseases such as cancer.

## Author contributions

The author confirms being the sole contributor of this work and approved it for publication.

### Conflict of interest statement

The author declares that the research was conducted in the absence of any commercial or financial relationships that could be construed as a potential conflict of interest.
